# Acute versus chronic methotrexate poisoning; a cross-sectional study

**DOI:** 10.1186/s40360-019-0316-8

**Published:** 2019-07-03

**Authors:** Arman Ahmadzadeh, Nasim Zamani, Hossein Hassanian-Moghaddam, Seyed Kaveh Hadeiy, Parinaz Parhizgar

**Affiliations:** 1 Department of Internal Medicine, Division of Rheumatology, Loghman Hakim Hospital, Shahid Beheshti University of Medical Sciences, Tehran, Iran; 2 Social Determinants of Health Research Center, Shahid Beheshti University of Medical Sciences, Tehran, Iran; 3 Department of Clinical Toxicology, Loghman Hakim Hospital, Shahid Beheshti University of Medical Sciences, South Karegar Street, Tehran, Iran; 4 School of Medicine, Shahid Beheshti University of Medical Sciences, Tehran, Iran

**Keywords:** Methotrexate, Poisoning, Toxicity, Acute, Chronic

## Abstract

**Background:**

Data is limited on comparison of acute and chronic methotrexate (MTX) poisoning. Methotrexate is an anti-folate drug that may be prescribed in some malignant or chronic inflammatory conditions. The aim of the current study was to compare signs and symptoms, complications, treatment and final outcome of acute and chronic MTX toxicity.

**Method:**

In a retrospective study in a referral center between March 2010 and March 2018, all patients who had been referred with the history of MTX poisoning and hospitalized due to acute or chronic poisoning were evaluated and compared.

**Results:**

Of the total 27 patients admitted during the study period, 13 had referred with acute (group 1; consumption of MTX for less than 7 days) and 14 had referred with chronic toxicity (group 2; consumption of MTX for more than 7 days). Mean age was significantly higher in the second group (*P* < 0.001). Median total dose of MTX was similar between the groups (*P* = 0.90). Mucosal ulcers and skin lesions (*P* < 0.001 and 0.02, respectively) were the only symptoms significantly different between the two groups. Leukopenia (*P* < 0.001), thrombocytopenia (*P* < 0.001), and anemia (*P* = 0.04) were significantly more common in the second group. Blood urea nitrogen and creatinine were also significantly higher in the second group of the patients (*P* < 0.001 and *P* = 0.048). Median leucovorin administered dose was 200 mg [14, 480] versus 150 mg [75, 187] (*P* = 0.69) in groups 1 and 2, respectively.

**Conclusions:**

Chronic MTX poisoning is more serious than acute toxicity and accompanies higher dermatologic, hematologic, and hepatic complications necessitating more aggressive treatments including administration of higher doses of leucovorin or bone marrow stimulants such as G-CSF. This may be attributable to the underlying diseases and features (including older ages) which predispose these patients to complications.

## Background

Methotrexate (MTX) is a folic acid analogue and antagonist used in the treatment of autoimmune diseases, blood and solid organ malignancies, dermatologic diseases, and termination of pregnancy [1, 2]. It inhibits dihydrofolate reductase interfering with the conversion of dihydrofolate to tetrahydrofolate which is the primary carbon donor for purine and pyrimidine synthesis [2].

Different side effects have been reported after MTX administration. Although they are more common after administration of high dose MTX (HDMTX; > 500 mg/m^2^), they have also been reported after lower doses. The complications of MTX poisoning include acute kidney injury, hepatotoxicity, mucositis, neurotoxicity, and myelosuppression [3].

Lo Vecchio and colleagues evaluated 13 cases with acute MTX poisoning, all of whom survived. None of their cases showed toxicity and the authors, therefore, concluded that acute poisoning either intentional or accidental was usually safe [2]. They also mentioned that this type of toxicity did not typically need leucovorin.

On the other hand, it has been reported that acute doses as low as 10 mg orally might lead to adverse effects and treatment until the blood methotrexate level is below 0.05 μmol/L is generally advocated [4]. However, in many cases MTX level is not available and the treatment regimen should be titrated based on some other endpoint.

Currently, basic recommendations for treating oral MTX overdose include administration of activated charcoal, gastric lavage, folinic acid, and urine alkalization depending on the severity of the toxicity [5]. However, the appropriate treatment to be given in the acutely and chronically poisoned patients with MTX intoxication remains unclear mainly due to the low prevalence of the poisoning. Although few studies have evaluated acute and chronic MTX toxicity, no study has compared them, to date. Rate of complications and outcome seem to be significantly different between these two groups of toxicity. There is no definite cut-off point from which we decide to treat patient’ complications specially in acute toxicities. Although watch and wait approach has been recommended for acutely MTX-poisoned patients, it seems that in some patients with more severe toxicities, more aggressive treatments are needed. The aim of the current study was to define signs and symptoms, complications, treatment and final outcome of acute MTX toxicity in comparison with a more clarified territory of chronic MTX poisoning.

## Methods

In a retrospective single-center study on the MTX-poisoned patients admitted to our referral center between March 2010 and March 2018, all patients who had been referred to our center with the history of MTX poisoning and hospitalized to either clinical toxicology department (acute cases) or rheumatology ward (chronic cases) were evaluated. In our center, cases of acute toxicity are admitted to the toxicology ward. However, patients who use medications for their background disorders and refer with complications of their treatments are admitted to the primary service managing them which was the rheumatology ward in this case. They were all referred themselves and no case of referral from other centers was present among them. We reviewed the literature for a clear definition of acute versus chronic MTX toxicity. Although signs and symptoms were classified to acute and chronic manifestations, no cut-off level of ingested dose of MTX or duration of use was defined to distinguish acute from chronic toxicity.

We considered those with acute single ingestions as acutely poisoned patients. Those with acute poisoning on chronic use were considered to be acutely poisoned, as well, because their signs and symptoms implied acute toxicity in most of these cases. All other patients including those who used methotrexate on a weekly basis for treatment of rheumatologic and dermatologic conditions and had accidentally used MTX more than the usual dose (for instance due to misunderstandings in the dose and intervals) were considered to be chronically poisoned.

Using this classification, patients who used MTX to attempt suicide and those who accidentally ingested it for the first time were considered as those with acute intoxication. Chronic MTX toxicity included MTX overdose in a wider duration, concomitant use of other myelosuppressive drugs, and hypofolate status, although all patients with chronic toxicity were using folate supplements 5–15 mg/week. Patients in the first group had been asked to perform follow-up evaluations seven and 14 days after discharge and refer to toxicology outpatient clinic if any abnormal lab test was detected in their follow-up lab exams. Patients with chronic toxicity had been referred to out-patient rheumatology clinic for further follow-up.

A self-made questionnaire containing information on patients’ demographic characteristics (age, gender, and weight), mean ingested or injected dose (weekly dose in chronic toxicity), background diseases (malignancy, rheumatoid arthritis [RA], systemic lupus erythematous [SLE], hepatitis, renal insufficiency, diabetes mellitus [DM], cardiovascular diseases, and psoriasis), MTX formulation (parenteral versus oral), cause of acute poisoning (suicidal versus accidental), cause of chronic poisoning (use of incorrect dose or medication such as ingestion of MTX instead of folic acid, wrong timing that caused ingestion or injection of repeated doses, folate deficiency, and simultaneous use of other myelosuppressive drugs such as azathioprine), cause of MTX administration in chronic cases, on-presentation signs and symptoms, lab tests, need for intensive care unit (ICU) admission, need for hemodialysis, treatments given, and final outcome of the patients as well as mechanism of death in non-survivors was filled for every single patient. This questionnaire was designed at the beginning of the study and was not a part of our standard care.

The data was analyzed using statistical package for social software (SPSS) version 21 using Kolmogorov Smirnov, t test, Mann Whitney U test and chi square test. A *P* value less than 0.05 was considered to be statistically significant.

## Results

Of the total 27 patients admitted during the study period, 13 had referred with acute (group 1) and 14 had referred with chronic (group 2) poisoning (Table 1). Three acute cases had overdosed on multiple drugs including MTX. Two patients were admitted to ICU. Mean (SD) age (range) was 24.7 ± 2.5 (2, 57) and 55.7 ± 14.8 (30, 76) years in the groups 1 and 2, respectively (*P* < 0.001). Seven (about 54%) acutely poisoned patients were children and adolescents. Median used MTX dose was 2.5 mg [2.5, 2.5] (2.5) and 2.5 mg [2.5, 50] (2.5, 1000) in groups 1 and 2, respectively (*P* = 0.11), and most of the patients had used 2.5-mg tablets. Median [IQR] (min, max) total dose was 50 [16.2, 106.2] (5, 125) mg versus 40 [16, 66] (10, 1000) mg in acute and chronic cases, respectively (*P* = 0.90). Median blood urea nitrogen (BUN), creatinine, white blood cell (WBC) count, hemoglobin, and platelet count were significantly different between the two groups (Table 2).

**Table 1:** Patients’ demographic characteristics

No.	Age range (year)	Sex	Diagnosis of the basal illness	Dose of MTX	Period of MTX usage before the event	Type of event	Other drugs
1^a^	60–69	_Male_	RA	Undetermined	Undetermined	LOC, skin and mucosal lesions	–
2	2–9	Male	Accidental	Undetermined	1 day	Nausea and vomiting	–
3	2–9	Female	Accidental	Undetermined	1 day	–	
4	60–69	Female	RA	15 mg	More than 6 months	Mucosal lesions, nausea, vomiting, diarrhea, abdominal pain	Folic acid and hydroxychlorocin daily regularly
5	60–69	Female	RA	70 mg/week instead of 10 mg/week	More than 6 months	Mucosal lesions	–
6	40–49	Male	Suicidal	50 mg	1 day	–	–
7	20–29	Female	Suicidal	2.5× undetermined number	1 day	Nausea and vomiting	–
8	30–39	Female	Psoriasis Suicidal (MDT)	5 mg	Undetermined	Nausea and vomiting, drowsiness	Multi drug toxicity (MTX + Respiridone)
9	50–59	Female	RA	2.5× undetermined number	10 days	Mucosal lesion, diarrhea, GI bleeding	–
10	10–19	Female	Suicidal	2.5× undetermined number	1 day	Nausea and vomiting	–
11	10–19	Female	Suicidal	87.5 mg	1 day	Nausea and vomiting	–
12	40–49	Female	Suicidal	125 mg	1 day	–	–
13	50–59	Female	RA Suicidal	150 mg	Undetermined	–	–
14	10–19	Male	Suicidal	75 mg	1 day	Nausea and vomiting	–
15	70–79	Female	RA	70 mg/week instead of 10 mg/week	11 days	Mucosal lesions	–
16	50–59	Female	Suicidal (MDT)	50 mg	1 day	–	MTX+ diazepam+ acetaminophen
17	2–9	Male	Accidental	20 mg	1 day	–	
18	10–19	Female	Suicidal	12.5 mg	1 day	–	MTX+ Prednisolone+ Azathioprine
19	40–49	Female	SLE	50 mg/week instead of 15 mg/week	Undetermined	Mucosal lesions	–
20	70–79	Female	RA	50 mg/week instead of 15 mg/week	More than 2 months	Mucosal lesions	–
21	30–39	Female	Psoriasis, SLE	10 mg weekly	More than 1 year	Skin and mucosal lesions, GIB	Azathioprine
22	30–39	Female	SLE	7.5 mg weekly	More than 6 months	Skin and mucosal lesions	Did not take the prescribed Folic acid
23	50–59	Female	RA	7.5 mg/day instead to 7.5 mg/week	Undetermined	Mucosal lesions, nausea, vomiting, diarrhea	Did not take the prescribed Folic Acid
24	50–59	Female	RA	50 mg weekly instead of 25 mg weekly	1.5 month	Skin and mucosal lesions, abdominal pain, GIB	–
25	40–49	Female	RA	Amp 1000 mg instead of 50 mg weekly	3 weeks	Skin and mucosal lesions, nausea and vomiting, abdominal pain	–
26	70–79	Female	RA	17.5 mg instead of 2.5 mg weakly	More than 6 months	Mucosal lesions, nausea, vomiting, diarrhea, abdominal pain	–
27	50–59	Female	RA	30 mg/week instead of 15 mg/week	More than 4 months	Skin and mucosal lesions	Cyclosporine

^a^The only case of mortality

**Table 2:** Comparison of the lab test results between patients with acute and chronic poisonings

	Acute (*n* = 13)	Chronic (*n* = 14)	P	Total (*n* = 27)
Median [IQR] Creatinine (mg/dL)	0.8 [0.7, 1.1]	1.0 [0.9, 1.4]	0.048	1.0 [0.8, 1.1]
(min, max)	(0.6, 1.2)	(0.7, 2.1)		(0.6, 2.1)
Median [IQR] BUN (mg/dL)	11 [10, 12]	21 [16, 35.7]	< 0.001	15 [11, 22]
(min, max)	[8, 15]	(13, 80)		[8, 80]
Mean Total bilirubin (SD) (mg/L)	0.9 ± 0.4	1.2 ± 0.7	0.304	1.04 ± 0.62
(min, max)	(0.5, 1.7)	(0.3, 2.9)		(0.3, 2.9)
Median [IQR] Direct bilirubin (mg/L)	0.20 [0.12, 0.20]	0.20 [0.12, 0.55]	0.238	0.20 [0.20, 0.4]
(min, max)	(0.1, 0.6)	(0.1, 0.6)		(0.1, 2.0)
Median [IQR] INR (IU)	1.0 [1.0, 1.1]	1.0 [0.9, 1.1]	0.272	1.0 [0.9, 1.1]
(min, max)	(0.9, 1.2)	(0.9, 2.0)		(0.9, 2.0)
Mean (SD) WBC (/mm^3^)	8313 ± 3761	2050 ± 1251	< 0.001	5066 ± 4181
(min, max)	(1480, 15200)	(400, 4400)		(400, 15,200)
Mean Hgb (SD) (mg/dL)	12.7 ± 2.1	9.1 ± 1.3	< 0.001	10.8 ± 2.5
(min, max)	(9.0, 16.7)	(6.5, 11.3)		(6.5, 16.7)
Mean Platelet (SD) (/mm^3^)	253846 ± 56388	78500 ± 39868	< 0.001	162925± 101161
(min, max)	(134000, 326000)	(35000, 147000)		(40000, 326000)
Mean PCo2 (SD) (mmHg)	39.6 ± 5.07	34.3 ± 8.56	0.093	37.1 ± 7.3
(min, max)	(28.0, 46.7)	(20.7, 45.0)		(20.7, 46.7)
Mean HCo3 (SD) (mEq/L)	24.8 ± 4.3	23.8 ± 5.0	0.627	24.4 ± 4.5
(min, max)	(19.3, 34.8)	(16.0, 30.7)		(16, 34)
Mean PTT (SD) (seconds)	31 ± 4	31 ± 6	0.077	30 ± 4.7
(min, max)	(25, 38)	(24, 40)		(24, 39)
Mean Urine (SD) pH*	6.4 ± 1.2	6.2 ± 1.0	0.714	6.3 ± 1.0
(min, max)	(5, 7.5)	(5, 7.5)		(5, 7.5)

*Subject to missing data

Patients with acute toxicity were generally those who had ingested their relatives’ medication to attempt suicide or those with accidental exposure to oral MTX. Four patients in this group were chronic MTX users due to RA and psoriasis who had attempted suicide. Underlying diseases including RA, psoriasis, DM, and SLE were present in 10, one, two, and three patients in the second group. All acutely poisoned patients had overdosed on oral form of MTX while in group 2, eight had ingested MTX and five had injected it. In this group, three, one, and five were poisoned due to incorrect dose consumption, wrong medication consumption, and incorrect time of consumption of the medication (daily instead of weekly). One case was poisoned because of both incorrect medication and incorrect dose. Two had folate deficiency (determined by increased MCV) and history of azathioprine use in the second group.

All 13 patients in group 1 had referred with the chief complaint of drug overdose (to toxicology emergency department) while in the second group, four patients had referred with cellulitis, fever and neutropenia, SLE flare-up, and weakness (one each). Another two patients in this group had referred with pancytopenia.

In acute cases, the most common symptoms on presentation were nausea and vomiting (6 cases, 46.2%) and one had referred with loss of consciousness (7.7%). In chronic cases, the most common manifestations were mucosal ulcers in 13 (92.8%) followed by skin lesions (in six), nausea and vomiting (in five), diarrhea (in four), abdominal pain (in four), GI bleeding (in three), and loss of consciousness (in one). Table 3 shows variables with significant difference between acute and chronic toxicity cases.

**Table 3:** Significant Risk factors determining odds of being acute vs. chronic Methotrexate toxicity (*n* = 27)

Characteristics	Acute (*n* = 13)	Chronic (*n* = 14)	*p* value	OR (95%CI)
RA Background disease n(%)	3 (23.1)	10 (76.9)	0.021	8.3 (1.4, 47.2)
MTX oral formulation	13 (61.9)	8 (38.1)	0.016	1.8 (1.1, 2.8)
MTX injection formulation	0	5 (100)	0.041	1.6 (1.1, 2.3)
Suicide Attempt n(%)	10 (100)	0	> 0.001	0.23 (0.07, 0.62)
Error in time of prescription drug n(%)	0	5 (100)	0.041	1.6 (1.1, 2.3)
Skin lesions n(%)	0	6 (100)	0.016	1.7 [1.1, 2.7)
Oral ulcerations n(%)	0	13 (100)	> 0.001	14 (2.1, 92.5)
Leukopenia n(%)	1 (7.7)	12 (92.3)	> 0.001	72 (5.7, 904.1)
Thrombocytopenia n(%)	1 (7.7)	14 (100)	> 0.001	15 (2.3, 99.6)
Anemia n(%)	9 (39.1)	14 (60.9)	0.041	0.69 (0.48, 0.99)

Mucosal ulcers and skin manifestations with odds ratios (95% CI) of 14 (2.1, 92.5) and 1.7 (1.1, 2.7) (*P* < 0.001 and 0.02, respectively) were the only symptoms significantly different between the two groups. Leukopenia (*P* < 0.001), thrombocytopenia (*P* < 0.001), and anemia (*P* = 0.04) were significantly more common in the second group with odds (95% CI) of 72 (5.7, 904.1), 15 (2.3, 99.6), and 0.69 (0.48, 0.99), respectively.

Seven and four patients in group one needed leucovorin and sodium bicarbonate while all patients in group two needed treatment with leucovorin (8 patients; 57.1%), sodium bicarbonate (2; 14.2%), Granulocyte-colony stimulating factor (GCSF; 5; 35.7%), packed cell (5; 35.7%), platelet (1; 7.1%), and fresh frozen plasma (FFP; 1; 7.1%).

Median leucovorin dose administered was 200 [14, 480] (12, 750) and 150 [75, 187] (30, 300) (*P* = 0.69) mg in groups 1 and 2, respectively. Median hospitalization period was 2 days [1, 3] (1,3) in acute cases and 6.5 days [5.5, 10.2] (3,27) in chronic cases (*P* < 0.001). One 61-year-old patient died in the second group due to pancytopenia and sepsis. Among chronic cases, the median hospitalization period was 6 [5, 11] (3, 22) in injection exposure and 8 [5, 11] (3, 27) in oral ingestions (*P* = not significant).

## Discussion

Methotrexate is a popular medication generally used to treat rheumatoid arthritis, psoriatic arthritis and vasculitis. It is generally considered to be safe and therefore, it is not unusual to prescribe doses as high as 25 to 30 mg per week in modern rheumatology [6, 7].

Methotrexate toxicity is mainly due to its effects on folate metabolism. It has also been suggested that MTX polyglutamates may cause hepatotoxicity by folate depletion although other mechanisms including inhibition of purine metabolism, adenosine deaminase with accumulation of adenosine and deoxyadenosine, polyamine synthesis, and homocysteine metabolism have also been suggested to explain the cause of MTX toxicity [8, 9].

The most common cause of MTX toxicity is accidental overdose or erroneous administration by the physician or pharmacist. Our results showed that suicidal attempt in chronic users is a major concern and should be considered in those who are depressed and have suicidal thoughts. On the other hand, most of the cases in acute toxicity were younger than 18 years and had attempted suicide, both of which confirm the danger of keeping MTX available.

Most of the chronic users had made a mistake distinguishing between MTX and folic acid tablets. For those who inject MTX, injections drawing higher doses of drug from a vial into the syringe can happen. The available injection forms of MTX are 50 mg/5 mL and 50 mg/2 mL for rheumatic disorders and 1000 mg/ 5 mL for chemotherapy in our pharmacopeia. Taking similar volumes from vials with different strengths is not an uncommon mistake and may result is toxicity.

Usually, a chronically MTX-poisoned patient is the one with a long-standing RA or psoriasis/psoriatic arthritis presenting with sudden onset of erosions or ulcers in psoriatic plaques and/or sudden onset of severe mucosal ulceration in the oral cavity with or without diarrhea and fever secondary to infection. This was according to our results and mucosal ulceration was seen in all chronic cases except one.

Some major predisposing factors for developing chronic MTX toxicity are older age, renal failure, hypoalbuminemia, and simultaneous use of drugs which interact with MTX including salicylates, nonsteroidal anti-inflammatory drugs (by decreasing renal excretion and tubular secretion of MTX), trimethoprim/sulfamethoxazole and sulfasalazine (by accentuating the cytotoxic effects of MTX via concomitant inhibition of dihydrofolate reductase), and leflunomide and azathioprine (by imposing myelosuppressive effects). Significant differences in age, blood urea nitrogen and creatinine confirmed the possible role of renal impairment in detoxification of chronic toxicity cases in the current study.

Since both dose and duration of MTX use may correlate with toxicity, measurement of serum levels of MTX may help predict (GI) toxicity and myelosuppression [10]. However, rapid disappearance of MTX from the serum within 24 h of administration impairs this ability significantly [11]. Therefore, none of the guidelines available on treatment of MTX poisoning recommends serum level measurements. These guidelines mostly focus on evidence of end organ damage [12]. We believe this is the cause of higher incident of invasive treatment in chronic cases. In fact, their older age, background kidney involvement, more common hematologic complications and more severe gastrointestinal (GI) symptoms made us treat chronically poisoned patients more aggressive.

Hepatotoxicity is a common complication of long-term treatment with MTX and is mostly expected in chronic toxicities [13]. Several cases of mild hepatotoxicity due to chronic use of MTX were visited in our outpatient rheumatology clinic who were treated by dose reduction or discontinuation of methotrexate. They were self-limited in nature and were not included in the current study.

Hematologic toxicity is a serious complication commonly observed with high-dose MTX [14]. We showed that a simple complete blood count evaluation, which can be easily performed in even small unequipped laboratories with low cost, could yield valuable indices associated with chronic MTX toxicity including leukopenia, high MCV (> 95 fL), thrombocytopenia, and anemia. Interestingly, we had a 3-year-old toddler with no history of MTX use who experienced pancytopenia after accidental ingestion of 20 mg MTX. His leukopenia resolved within 3 days with supportive care and without need for G-CSF.

Thrombocytopenia followed by a rapidly progressive leuko-neutropenia is another feature in chronic toxicity [15]. Hematologic toxicity including thrombocytopenia, megaloblastic anemia, leukopenia and pancytopenia are rare with low doses of the medication and can both happen in 2 weeks after the acute overdose or in chronic uses to treat rheumatoid arthritis and psoriaisis [16]. In our patients, frequency of hematologic complications including anemia, thrombocytopenia, and leukopenia was significantly higher in chronically poisoned patients. We had no report of such complications in our out-patient clinic, where the acute poisoning cases were advised to refer for follow-up. This is in accordance with previous reports considering saturable absorption of MTX bioavailability in acute overdoses [17].

The most frequent mucocutaneous reactions to MTX are ulcerations of the oral mucosa, burning sensation of the skin, photosensitivity, acral erythema, multiform erythema, urticaria and vasculitis [18]. Skin lesions occur within the first week of treatment, continue to evolve in the second week and last for 4 to 7 days [19]. About 93% of our patients in group 2 had signs/symptoms of mucositis and oral ulcers while none in group 1 had such symptoms. However, nausea/vomiting was more prevalent in the acute toxicity group (*P* = not significant). Other GI symptoms including abdominal pain, diarrhea, and GI bleeding were more prevalent in chronic cases.

Although nephrotoxicity is more common with doses higher than 500 mg/m^2^ and therefore more expected in acute toxicities, both BUN and creatinine were significantly higher in chronic cases. This again can be attributed to chronic underlying diseases of the patients in group 2 and should be considered in all patients on chronic use of MTX.

All patients in group 2 needed treatment with administration of one or more medications; this is while only seven and four in acute cases needed leucovorin and sodium bicarbonate. None of these patients needed GCSF, packed cell, platelet, or FFP infusion while five, four, one, and one patients in the second groups needed the above-mentioned treatments, respectively. This emphasizes on the self-limited nature of acute MTX toxicity compared to the chronic poisoning although in some cases treatment is warranted in acute cases, as well.

## Limitations

The limited number of our patients as well as the retrospective nature of the study are definitely the major limitations of the current study. Another major limitation is the lack of a definite criteria in the current literature to distinguish chronic from acute MTX toxicity while these two types of toxicity require different approaches to be treated. In acute cases, our follow-up was based on advice given to the patients to refer to outpatient clinics mainly due to the fact that we could not keep the patients in hospital for 14 days. We had some suicidal patients who refused to be followed up. We have no data on possible adverse effects if the patients have developed complications and referred to other health care facilities. Also, we could not check the serum level of MTX which limits our results. Future prospective studies should be run on more cases to clarify the features of acute and chronic MTX poisoning.

## Conclusion

Complications are different in acute and chronic MTX poisonings. Different organs may be involved in these two entities. Thus, discrete treatment approaches must be taken with consideration of the type of toxicity. Chronic MTX poisoning is more serious and accompanies higher muco-cutaneous, hematologic, and hepatic complications necessitating more aggressive treatments including administration of higher doses of leucovorin or bone marrow stimulants including G-CSF. Lack of a clear definition for acute and chronic MTX poisoning is a major limitation in rheumatologic and toxicologic textbooks and further tries should be made to distinguish these two phenomena. Care should be give when administering different different-strength doses of MTX to avoid chronic toxicity. This can be done by developing electronic prescriptions which automatically prevent dose-dependent errors by pharmacists, warning or limiting the pharmacist access to chemotherapy doses for rheumatologic patients, or through giving advice regarding the MTX doses for each patient. Keeping MTX tablets out of the reach of the children and toddlers is another major recommendation to prevent further accidental MTX intoxications in this age group.

## Data Availability

All data analysed during this study are included in this published article.

## Abbreviations

BUN: Blood Urea Nitrogen
CI: Confidence Interval
DM: Diabetes Mellitus
FFP: Fresh Frozen Plasma
G-CSF: Granulocyte - Colony Stimulating Factor
GI: Gastro-Intestinal
HDMTX: High Dose Methotrexate
ICU: Intensive Care Unit
MCV: Mean Corpuscular Volume
MTX: Methotrexate
RA: Rheumatic Arthritis
SD: Standard Deviation
SLE: Systemic Lupus Erythematous
SPSS: Statistical Package for Social Software
WBC: White Blood Cell
